# The *pla* gene, encoding plasminogen activator, is not specific to *Yersinia pestis*

**DOI:** 10.1186/s13104-015-1525-x

**Published:** 2015-10-05

**Authors:** Stephanie Hänsch, Elisabetta Cilli, Giulio Catalano, Giorgio Gruppioni, Raffaella Bianucci, Nils C. Stenseth, Barbara Bramanti, Mark J. Pallen

**Affiliations:** Centre for Ecological and Evolutionary Synthesis, University of Oslo, Oslo, Norway; Department of Cultural Heritage, University of Bologna, Ravenna Campus, Bologna, Italy; Microbiology and Infection Unit, Warwick Medical School, University of Warwick, Coventry, CV4 7AL UK

## Abstract

Here we present evidence to show that the *pla* gene, previously thought to be specific to *Yersinia pestis*, occurs in some strains of *Citrobacter koseri* and *Escherichia coli*. This means that detection of this gene on its own can no longer be taken as evidence of detection of *Y. pestis*.

## Correspondence

Molecular assays aimed at detecting traces of the etiological agent of plague, *Yersinia pestis*, have focused primarily—and sometimes solely—on the plasminogen activator/coagulase (*pla*) gene [1, 2]. This gene is located on the pPCP1 plasmid and has been considered the target of choice for plague detection due to its assumed specificity to *Y. pestis* and its occurrence in multiple copies [3–5]. However, a recent paper about the amplification of the *pla* gene from tissues from European rats has raised doubts over whether this gene is indeed specific to *Y. pestis* [6]. We can now confirm this suspicion.

We screened archaeological samples from Italy (6th, 14th and 17th centuries CE), amplifying a 70-base-pair fragment from the *pla* gene. Full protocols are described in a previous publication [7], but in brief we performed the work in a dedicated clean laboratory, with physically separated areas for extraction and amplification, following the most stringent criteria for ancient DNA analysis, such as the use of mock extractions and PCR blanks. We used the previously described *pla* primer pair (Forward primer: GACTGGGTTCGGGCACATGC—Reverse primer: CGGATGTCTTCTCACGGA). Cycling conditions started with an initial activation step at 95 °C for 15 min. This was followed by 50 cycles at 94 °C for 30 s, an assay specific annealing temperature at 60 °C for 30 s, and 72 °C for 1 min, ending with a final elongation step at 72 °C for 10 min. Final cooling was carried out at 8 °C until analysis.

**Fig. 1:**
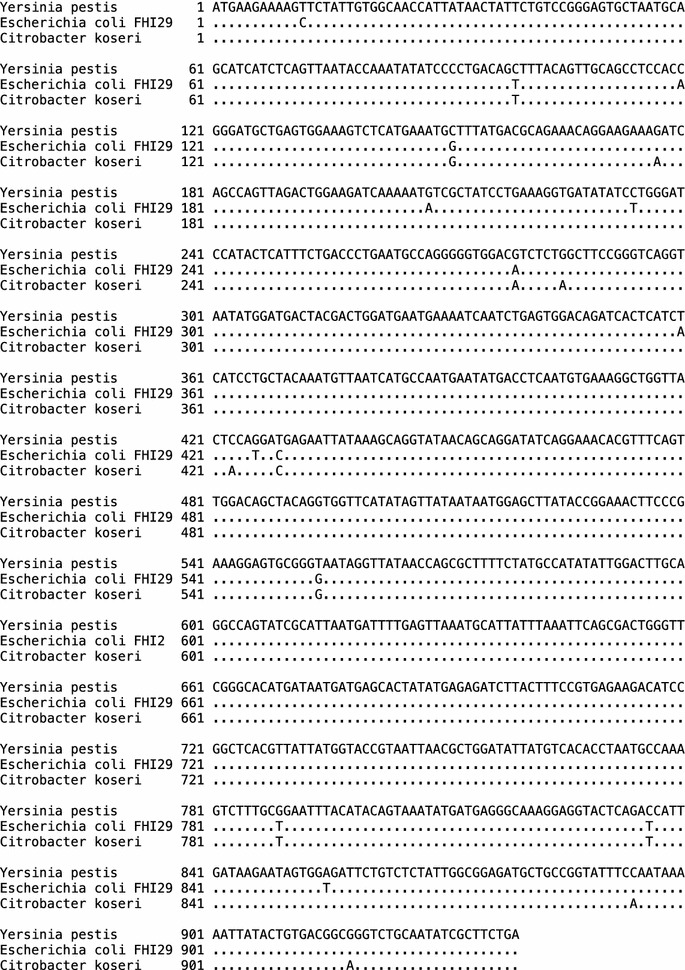
Multiple alignment of *pla* sequences from *Yersinia pestis* and two other species

Target-fragment amplifications were observed in 17 out of 40 samples. We sequenced one of the fragments and performed a BLASTN search of the NCBI database, which, as expected, revealed full-length identity (70/70 at nucleotide level) with numerous sequences from *Y. pestis*. However, we also found full-length identity with two sequences from outside *Y. pestis*. The first was an annotated contig from a genome assembly of *Citrobacter koseri* (submitted to GenBank in June 2014, with accession number LK931337). This bacterial species has been recognized as a commensal and pathogen in humans and animals [8, 9]. The second was from a contig from an unannotated genome assembly of *Escherichia coli* strain FHI29 (submitted to GenBank in June 2014, with accession number LM995843). This sequence is derived from a human fecal isolate from a case of gastroenteritis in Norway. To conduct a more extensive survey, we then performed BLAST searches with the entire *pla* gene from *Y. pestis* CO92, which confirmed the high level of similarity (>98 %) with sequences from the genome assemblies of *C. koseri* (927/939 identities at nucleotide level) and of the *E. coli* strain FHI29 (925/939 identities) (Fig. 1). The relevant contig from *C. koseri* contained sequences annotated with plasmid-related functions, suggesting that the *pla* gene in this context is also plasmid-encoded.

The presence of *pla* sequences from outside *Y. pestis*, each derived from a distinct geographical or taxonomic setting, confirms beyond doubt that this gene can no longer be considered specific to *Y. pestis*. Although there appear to be some potentially informative sequence differences between the *pla* sequences from *Y.pestis* and those from other taxa, these findings call into question any results—whether in contemporary diagnostic microbiology or in an ancient DNA setting—that rely on detection of PCR products from this gene alone. Instead, as many researchers in the field already recognise, it is important to obtain sequences from PCR products and detection or identification of *Y. pestis* should rely on sequences from at least two independent molecular targets. More generally, our observations call into question the wisdom of relying on genes from mobile elements as species-specific markers, given the likelihood that such sequences are able to move from one taxon to another. Interestingly, the roles of the sequence differences between the pla genes, some of which are non-synonymous, in the function and evolution of the pla gene product remain to be determined.
